# Comparative Analyses of Fundamental Differences in Membrane Transport Capabilities in Prokaryotes and Eukaryotes

**DOI:** 10.1371/journal.pcbi.0010027

**Published:** 2005-08-19

**Authors:** Qinghu Ren, Ian T Paulsen

**Affiliations:** The Institute for Genomic Research, Rockville, Maryland, United States of America; EMBL Heidelberg, Germany

## Abstract

Whole-genome transporter analyses have been conducted on 141 organisms whose complete genome sequences are available. For each organism, the complete set of membrane transport systems was identified with predicted functions, and classified into protein families based on the transporter classification system. Organisms with larger genome sizes generally possessed a relatively greater number of transport systems. In prokaryotes and unicellular eukaryotes, the significant factor in the increase in transporter content with genome size was a greater diversity of transporter types. In contrast, in multicellular eukaryotes, greater number of paralogs in specific transporter families was the more important factor in the increase in transporter content with genome size. Both eukaryotic and prokaryotic intracellular pathogens and endosymbionts exhibited markedly limited transport capabilities. Hierarchical clustering of phylogenetic profiles of transporter families, derived from the presence or absence of a certain transporter family, showed that clustering patterns of organisms were correlated to both their evolutionary history and their overall physiology and lifestyles.

## Synopsis

Membrane transporters are the cell's equivalent of delivery vehicles, garbage disposals, and communication systems—proteins that negotiate through cell membranes to deliver essential nutrients, eject waste products, and help the cell sense environmental conditions around it. Membrane transport systems play crucial roles in fundamental cellular processes of all organisms. The suite of transporters in any one organism also sheds light on its lifestyle and physiology. Up to now, analysis of membrane transporters has been limited mainly to the examination of transporter genes of individual organisms. But advances in genome sequencing have now made it possible for scientists to compare transport and other essential cellular processes across a range of organisms in all three domains of life.

Ren and Paulsen present the first comprehensive bioinformatic analysis of the predicted membrane transporter content of 141 different prokaryotic and eukaryotic organisms. The scientists developed a new computational application of the phylogenetic profiling approach to cluster together organisms that appear to have similar suites of transporters. For example, a group of obligate intracellular pathogens and endosymbionts possess only limited transporter systems in spite of the massive metabolite fluxes one would expect between the symbionts and their host. This is likely due to the relatively static nature of their intracellular environment. In contrast, a cluster of plant/soil-associated microbes encode a robust array of transporters, reflecting the organisms' versatility as well as their exposure to a wide range of different substrates in their natural environment.

## Introduction

Membrane transport systems play essential roles in cellular metabolism and activities. Transporters function in the acquisition of organic nutrients, maintenance of ion homeostasis, extrusion of toxic and waste compounds, environmental sensing and cell communication, and other important cellular functions [1]. Various transport systems differ in their putative membrane topology, energy coupling mechanisms, and substrate specificities [2]. Among the prevailing energy sources are adenosine triphosphate (ATP), phosphoenolpyruvate, and chemiosmotic energy in the form of sodium ion or proton electrochemical gradients.

The transporter classification system (http://www.tcdb.org/) represents a systematic approach to classify transport systems according to their mode of transport, energy coupling mechanism, molecular phylogeny, and substrate specificity [2–5]. Transport mode and energy coupling mechanism serve as the primary basis for classification because of their relatively stable characteristics. There are four major classes of solute transporters in the transporter classification system: channels, primary (active) transporters, secondary transporters, and group translocators. Transporters of unknown mechanism or function are included as a distinct class. Channels are energy-independent transporters that transport water, specific types of ions, or hydrophilic small molecules down a concentration or electrical gradient; they have higher rates of transport and lower stereospecificity than the other transporter classes (e.g., *Escherichia coli* GlpF glycerol channel [6]). Primary active transporters (e.g., *Lactococcus lactis* LmrP multidrug efflux pump [7]) couple the transport process to a primary source of energy (ATP hydrolysis). Secondary transporters utilize an ion or solute electrochemical gradient, e.g., proton/sodium motive force, to drive the transport process. *E. coli* LacY lactose permease [8,9] is probably one of the best characterized secondary transporters [10]. Group translocators modify their substrates during the transport process. For example, *E. coli* MtlA mannitol PTS transporter phosphorylates exogenous mannitol using phosphoenolpyruvate as the phosphoryl donor and energy source and releases the phosphate ester, mannitol-1-P, into the cell cytoplasm [11,12]. Each transporter class is further classified into individual families and subfamilies according to their function, phylogeny, and/or substrate specificity [3].

Since the advent of genomic sequencing technologies, the complete sequences of over 200 prokaryotic and eukaryotic genomes have been published to date, representing a wide range of species from archaea to human. There are also more than 1,100 additional genome sequencing projects currently underway around the world (Gold Genomes Online Database, http://www.genomesonline.org/) [13,14]. Convenient and effective computational methods are required to handle and analyze the immense amount of data generated by the whole-genome sequencing projects. An in-depth look at transport proteins is vital to the understanding of the metabolic capability of sequenced organisms. However, it is often problematic to annotate these transport proteins by current primary annotation methods because of the occurrence of large and complex transporter gene families, such as the ATP-binding cassette (ABC) superfamily [15,16] and the major facilitator superfamily (MFS) [17,18], and the presence of multiple transporter gene paralogs in many organisms. We have been working on a systematic genome-wide analysis of cellular membrane transport systems. Previously, we reported a comprehensive analysis of the transport systems in 18 prokaryotic organisms [19,20] and in yeast [21]. Here we expand our analyses to 141 species and compare the fundamental differences in membrane transport systems in prokaryotes and eukaryotes. Phylogenetic profiling of transporter families and predicted substrates was utilized to investigate the relevance of transport capabilities to the overall physiology of prokaryotes and eukaryotes.

## Results/Discussion

### Numbers of Recognized Transporter Families and Proteins

**Figure 1:**
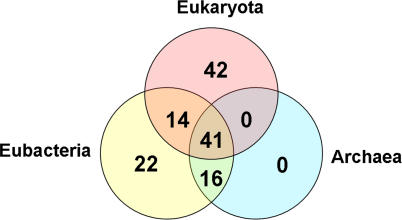
Venn Diagram Showing the Distribution of Transporter Families across the Three Domains of Life

A total of 40,678 transport proteins from 141 species (Table S1), including 115 Eubacteria, 17 Archaea, and 9 Eukaryota, were predicted by our analysis pipeline. They were classified into 134 families, including 7 families of primary transporters, 80 families of secondary transporters, 32 channel protein families, 2 phosphotransferase systems (PTSs), and 13 unclassified families. Some of these families are very large superfamilies with numerous members, such as the ABC superfamily and MFS, both of which are widely distributed in Eubacteria, Archaea, and Eukaryota. Some are small families with only a single or a few members. The distribution of transporter families varies significantly across the three domains of life (Figure 1). There are 42 eukaryotic-specific families, mostly ion channel families that exist exclusively in multicellular eukaryotic organisms like *Drosophila melanogaster,* *Arabidopsis thaliana,* and humans. These channels are involved in processes like cell communication, signal transduction, and maintenance of internal homeostasis in a multicellular environment. Most of these families are restricted to a single organismal type. Many of them may have arisen later during evolution, after the separation of the three domains. Alternatively, some families may have diverged too extensively from their prokaryotic counterparts to be recognized as homologs. Interestingly, a bacterial homolog to the previously described “eukaryotic-specific” glutamate-gated ion channel (GIC) family of neurotransmitter receptors has now been characterized in *Synechocystis* [22,23], and its orthologs have been identified in other sequenced Cyanobacteria. The *Synechocystis* transporter binds glutamate and forms a K^+^-selective ion channel. These observations suggest that eukaryotic GIC family transporters arose from a primordial prokaryotic counterpart.

There are 38 prokaryotic-specific transporter families, of which 22 families exist exclusively in Eubacteria, such as the bacterial sugar PTS systems (see below), and 16 are shared by Eubacteria and Archaea. In contrast to eukaryotic-specific families, which are usually limited to single species, the majority of prokaryotic-specific ones are broadly distributed among prokaryotes. There are no Archaea-specific transporter families currently known. Due to the very limited experimental characterization of Archaea species relative to Eubacteria and Eukaryota, many aspects of the physiology and biochemistry of Archaea are poorly understood [24]. We compared the annotation of membrane proteins in selected species of Archaea and Eubacteria in The Institute for Genomic Research's Comprehensive Microbial Resource database [25]. The percentage of the membrane proteins assigned to the role category of “hypothetical proteins” is significantly greater in Archaea than in Eubacteria (Figure S1). These observations suggest that the sparse functional characterization could be the primary reason for the lack of any known Archaea-specific transporter families.

There are 41 transporter families represented in all three domains of life, highlighting the fundamental importance of these families. These are presumably very ancient families shared by the last common ancestor of Archaea, Eukaryota, and Eubacteria. Most of them were found within the secondary transporter class. These ubiquitous transporter families function in the transport of a diverse spectrum of substrates, including sugars, amino acids, carboxylates, nucleosides, and various cations and anions. There are 14 families shared by Eubacteria and Eukaryota and 16 shared by Eubacteria and Archaea. Some of these families shared only in two domains may ultimately be discovered in all three domains once a greater diversity of organisms is sequenced.

**Figure 2:**
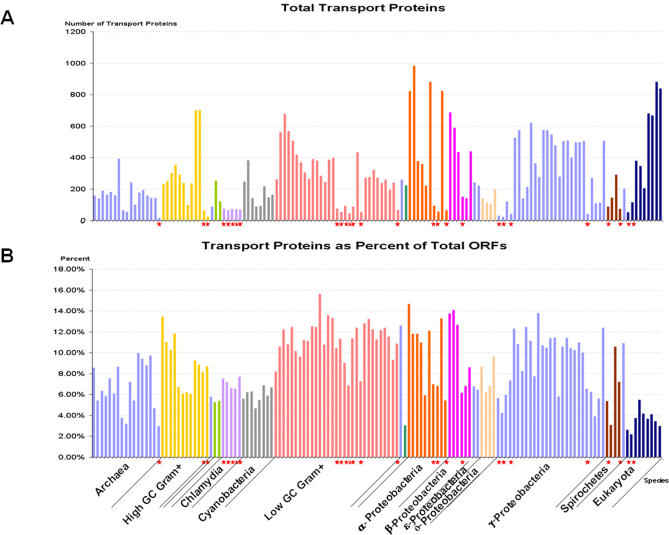
Numbers of Recognized Transport Proteins and Percentage of Total ORFs The overall numbers of recognized transport proteins (A) and percentage of total ORFs encoding transport proteins (B) were compared for the 141 organisms analyzed. Species from distinct phylogenetic groups are labeled with different colors. The prokaryotic and eukaryotic obligate intracellular parasites/pathogens are marked with red stars.

The overall quantity of recognized transport proteins (Figure 2A) and the percentage relative to the total number of open reading frames (ORFs) (Figure 2B) were compared for the organisms analyzed. Between 2% and 16% of ORFs in prokaryotic and eukaryotic genomes were predicted to encode membrane transport proteins, emphasizing the importance of transporters in the lifestyles of all species. In general, eukaryotic species, especially multicellular eukaryotic organisms, exhibit the largest total number of transport proteins, e.g., *Drosophila* (682 transport proteins, 3.7% of ORFs), *Arabidopsis* (882, 3.5%)*, Caenorhabditis elegans* (669, 4.1%), and humans (841, 3.0%). However, the transport proteins of eukaryotic species account for a relatively smaller percentage of total ORFs than in Eubacteria (average 9.3% ± 2.9%) and Archaea (average 6.7% ± 2.3%) species. Considerable variations in the quantity of transport proteins have been observed among species belonging to the same phylogenetic group. For example, α-Proteobacteria species exhibit a wide variety of lifestyles and corresponding differences in transporter content; they range from rhizosphere-dwelling organisms such as *Mesorhizobium loti* and *Sinorhizobium meliloti* [26] with 883 (12.1%) and 826 (13.3%) transport proteins each, to obligate intracellular pathogens or symbionts such as *Rickettsia prowazekii* and *Wolbachia* sp. with 57 (6.8%) and 65 (5.4%) transport proteins, respectively. Overall, prokaryotic obligate endosymbionts and intracellular pathogens, as well as the eukaryotic intracellular parasites (*Plasmodium falciparum* [27] and *Encephalitozoon cuniculi* [28]), possess the most limited repertoire of membrane transporters.

### Genome Size versus Diversity of Transporter Families and Numbers of Paralogs

**Figure 3:**
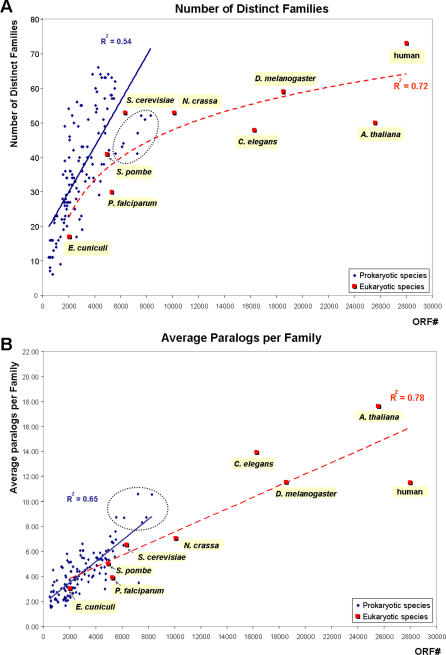
Number of Total ORFs versus Number of Distinct Transporter Families or Average Number of Paralogs per Family The number of total ORFs in the genome for each of the 141 sequenced prokaryotic and eukaryotic organisms (*x*-axis) was plotted as a function of either the number of distinct transporter families (A) or the average number of paralogs per family (B) (*y*-axis). Blue diamonds represent prokaryotic organisms and red squares represent eukaryotic organisms. Trend line and power correlation *R* ^2^ value are shown for prokaryotes and eukaryotes, respectively. A group of α-Proteobacteria are enclosed by a circle (see text for discussion).

Organisms with a larger genome size and therefore more ORFs generally encode a greater number of transporters [19,29]. In addition to transporters, regulatory genes, secondary metabolism genes, and transcription factors, also appear to increase with genome size [29–31]. Two major factors could contribute to the expansion of transporters in organisms with large genome sizes: (1) an increased number of distinct transporter families, and (2) a higher degree of gene duplication or expansion, leading to a greater number of paralogs in certain transporter families. To investigate the relationship between genome size and these two factors, we plotted the total number of ORFs from 141 organisms as a function of either the number of distinct transporter families (Figure 3A), or the average number of paralogs per family (Figure 3B). Prokaryotes and eukaryotes exhibit distinct differences. For prokaryotic species, there is a relatively linear relationship between the genome size and the number of transporter families (*R* ^2^ = 0.54) or average number of paralogs (*R* ^2^ = 0.65). As genome size increases, the rate of increase in the number of families per organism is approximately eight times greater than that of the average number of paralogs per family. The increase in genome size can only partially explain the expansion of transporter families and paralogs (as indicated by the correlation *R* ^2^ value). The strain-specific properties and lifestyles could also have an impact. For example, a group of α-Proteobacteria exhibit the most paralogs per family but have relatively lower diversity of transporter families. These organisms include rhizobial microsymbionts *M. loti,* *S. meliloti,* and *Bradyrhizobium japonicum* [26], and a closely related plant pathogen, *Agrobacterium tumefaciens* (enclosed by a circle on Figure 3). All of these organisms have more ABC transporters than any other sequenced organisms [29]. ABC family transporters mediate the uptake of a variety of nutrients and the extrusion of drugs and metabolite wastes. Having a large complement of high-affinity ABC uptake systems may be an advantage for organisms in the competition among microbes for nutrients. Two *Streptomyces* species, *St. avermitilis* and *St. coelicolor,* also exhibit a similar trend, with a significant expansion of the ABC and MFS family transporters.

The number of eukaryotic species analyzed is smaller, so it is more difficult to draw robust conclusions. The single-celled eukaryotes such as the yeasts appear to display characteristics similar to those of the prokaryotes, showing expansions in both transporter families and paralogs as genome size increases, with the former being a more important factor. However, in multicellular eukaryotic organisms such as animals and plants, the tremendous number of paralogs in certain transporter families accounts for a significant portion of the increase of transporters. Although multicellular eukaryotes exhibit fewer transporter families than some of the prokaryotic species, they have generated an extraordinary number of paralogs by gene duplication or expansion within certain families, like the ABC superfamily, MFS, and the voltage-gated ion channel superfamily. For example, the *Arabidopsis* genome encodes 110 paralogs of the ABC superfamily [32,33] and 92 paralogs of the MFS.

These differences in the relative abundances of transporter paralogs and distinct transporter families probably represent fundamental differences in transporter needs or priorities of these organisms. Multicellular organisms with many apparently redundant transporter paralogs appear to be utilizing a strategy of specialization. Many of their closely related paralogous transporters are presumably expressed only in specific tissues or subcellular localizations, or at specific developmental time points. Many appear to be involved in cell–cell communication and signal transduction processes, emphasizing the importance of intercellular communication in complex multicellular organisms. In contrast, the single-celled prokaryotes and eukaryotes, with relatively fewer paralogs but a greater emphasis on numbers of different families of transporters, appear to be utilizing a strategy of diversification. This probably reflects that one of the primary roles of membrane transport systems in these organisms is nutrient acquisition. A greater diversity of transporter types presumably allows for a broader range of substrate utilization.

### Distribution of Transporter Types According to Energy Coupling Mechanism

A wide range of variations were observed in the relative usage of energy coupling mechanisms to drive transport processes among the prokaryotes and eukaryotes analyzed. Table 1 shows the relative percentage of each transporter type in organisms from major phylogenetic groups. Transporters were categorized into five major types according to transport mode and energy coupling mechanism: primary transporters, secondary transporters, ion channels, group translocators, and unclassified. Primary and secondary carriers are ubiquitous, being present in all organisms analyzed. However, their percentage among the total transporters varies greatly (12%–78% for primary carriers and 17%–80% for secondary carriers). In prokaryotic and unicellular eukaryotic systems, primary and secondary carriers are the predominant types of transporters, together contributing more than 90% of the total transporters. Channel proteins make up a greater percentage (12%–43%) in higher eukaryotic organisms.

**Table 1:**
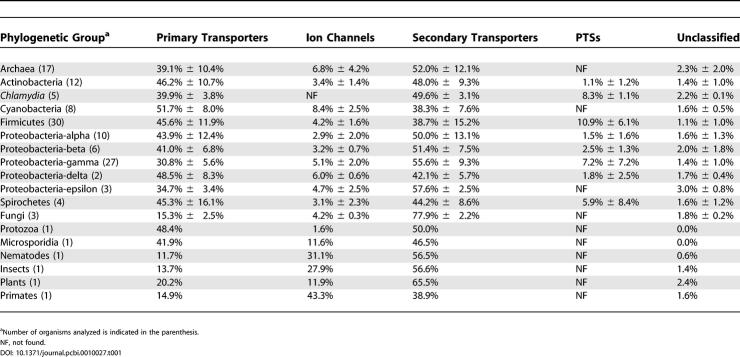
The Relative Percentage of Each Transporter Type within Major Phylogenetic Groups

^a^Number of organisms analyzed is indicated in the parenthesis.

NF, not found.

Compared to eukaryotes, prokaryotic organisms rely heavily on primary active transporters, largely because of the usage of ABC uptake systems that are absent in eukaryotes [34]. Organisms with the highest percentage of primary transporters generally belong to one of the three groups. (1) The first group includes organisms that lack a citrate cycle and an electron transfer chain, and therefore can only generate a proton motive force by indirect methods such as substrate-level phosphorylation followed by ATP hydrolysis. These organisms include *Mycoplasma* spp., spirochetes, *Streptococcus* spp., *Tropheryma whipplei,* *Mycobacterium leprae, Thermoanaerobacter tengcongensis,* and *Thermotoga maritime*. ATP is their primary source of energy, and therefore is most frequently used to drive nutrient uptake and maintain ion homeostasis. (2) The second group includes photosynthetic organisms with the ability to synthesize an ATP pool via photosynthesis, including *Synechocystis* sp., *Nostoc* sp., and *Thermosynechococcus elongates*. (3) The third group is a group of α-Proteobacteria that possess a significant expansion of the ABC superfamily [29], including soil/plant-associated bacteria, such as *M. loti* [26], *S. meliloti* [26], *A. tumefaciens,* and related human/animal pathogens such as *Brucella suis.* Unlike the first two groups, in which the usage of primary transporters seems to be predicated on bioenergetic constraints, the expansion of the ABC transporter family in these α-Proteobacteria does not have any obvious energetic explanations. Instead, it may reflect an organismal requirement for high-affinity transport since ABC transporters typically show higher substrate affinities than most secondary transporters.

The PTS is only present in a subset of Eubacteria, while completely lacking in Archaea and Eukaryota. Gram-negative enteric bacteria, such as *E. coli,* *Shigella flexneri,* and *Salmonella typhimurium*, as well as Gram-positive species associated with the human gastrointestinal tract, like *Listeria monocytogenes* and *Lactobacillus plantarum,* encode the most abundant PTS systems. Owing to the absorption capacity and efficiency of the intestine, these species have to compete with hundreds of other types of bacteria in an environment containing only small amounts of free carbohydrates or other easily absorbable forms of nutrients. The enrichment of sugar PTS systems in these species could be an advantage to thrive in their ecological niches.

Channel proteins contribute a relatively smaller percentage of transporters in the prokaryotic species we analyzed, and their functions in vivo are largely unknown. Nine organisms lack recognizable channels, including *Chlamydia* spp., *T. whipplei,* *Treponema pallidum,* *Wolbachia* sp., and *R.* *prowazekii,* all of which are obligate intracellular pathogens/symboints. All other prokaryotic species, including all extremophiles sequenced to date, encode channel proteins, suggesting these channels could function in responding promptly to osmotic and other environmental stresses [35]. Intracellular pathogens and endosymbionts may not need water or ion channels because of their relatively static intracellular environment and may largely depend on their host organisms for maintenance of ion homeostasis.

The percentage of channel proteins increases significantly in multicellular eukaryotes. In animals, these consist largely of ion channels with communication roles, such as in signal transduction, or roles as sensors for external stimuli. For example, members in the ligand-gated ion channel family [36] and the GIC family [37] are activated by major excitatory (glutamate) and inhibitory neurotransmitters (GABA) and participate in neuronal communication in the brain [38]. Recent studies show that some subunits of ligand-gated ion channels and GIC-type channels are expressed prominently during embryonic and postnatal brain development, while others are expressed mainly in the adult brain, suggesting that a switch in subunit composition may be required for normal brain development [38]. In plants, approximately one-third of the channel proteins are aquaporins (water channels) [39], many of which show a cell-specific expression pattern in the root, emphasizing the importance of regulating and maintaining turgor pressure through the plant [40].

Three fungal species, *Saccharomyces cerevisiae, Schizosaccharomyces pombe,* and *Neurospora crassa,* possess the largest portion of secondary transporters (76%–80%), mainly because of the prominent gene expansion of two types of functionally diverse MFS family transporters: (1) drug efflux pumps, which could play roles in the secretion of secondary metabolites, toxic compounds, and signaling molecules, and (2) sugar symporters, which could allow a broader range of sugar utilization [41,42].

### Phylogenetic Profiling of Transporter Family and Substrate Shows Strong Correlations to Organisms' Overall Physiology

The phylogenetic profile of a given protein is a string that encodes the presence or absence of that protein in every fully sequenced genome. Proteins that function together in a pathway or a common structural complex are likely to evolve in a correlated fashion, and therefore tend to be either preserved or eliminated together in a new species during evolution [43,44]. Phylogenetic profiling has been an effective way to detect conserved core genes, species-specific gene families, lineage-specific gene family expansions [45], and subcellular localization of proteins [46]. It can also facilitate the prediction of physical and functional interactions and assist in the deduction of the functions of genes that have no well-characterized homologs [47,48].

**Figure 4:**
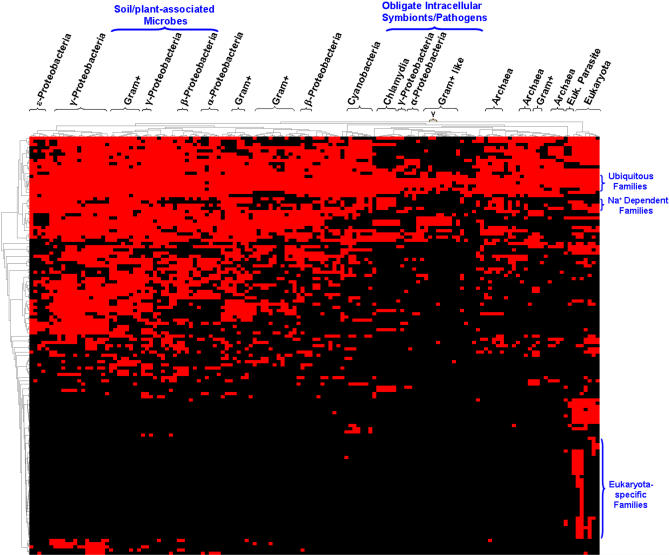
Phylogenetic Profiling of Transporter Families Phylogenetic profiles were created for each transporter family. Each profile is a string with 141 entries (number of organisms analyzed). If a given family is present in an organism, the value one is assigned at this position (red). If not, zero is assigned (black). Organisms and transporter families were clustered according to the similarity of their phylogenetic profiles.

**Figure 5:**
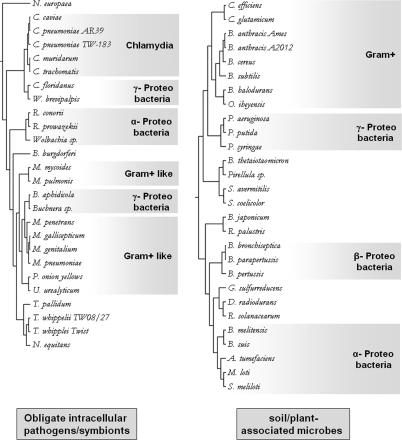
Hierarchial Clustering of Phylogenetic Profiles of Obligate Intracellular Pathogens/Symbionts versus Soil/Plant-Associated Microbes Detailed view of two clusters of organisms generated by hierarchical clustering of their phylogenetic profiles of transporter families: obligate intracellular pathogens/symbionts and soil/plant-associated microbes.

We have undertaken a novel application of phylogenetic profiling to investigate the presence or absence of transporter protein families across sequenced genomes. To our knowledge this represents the first application of a phylogenetic profiling approach using protein families rather than individual proteins as the unit of comparison. With the data on membrane transport systems from 141 fully sequenced organisms, we were able to construct the phylogenetic profiles for each transporter family (Figures 4 and S2). Hierarchical clustering of phylogenetic profiles showed a strong correlation between the observed clustering pattern and phylogeny, with Eubacteria, Archaea, and Eukaryota clearly separated into different clusters. Inside the bacterial cluster, Gram-positive bacteria, Proteobacteria, *Chlamydia,* and Cyanobacteria are also clearly defined into different groups. Given that the profiling approach solely utilizes presence or absence of a transporter family and does not use sequence similarity directly, this indicates that the types of transporters utilized by organisms are related to their evolutionary history. Additionally, the clustering appears to be influenced by habitat or lifestyle of organisms. For example, the obligate intracellular pathogens/symbionts and a collection of soil/plant-associated microbes are separated into two distinct superclusters (Figure 5).

The obligate intracellular pathogens/symbionts cluster includes a group of phylogenetically diverse organisms, including *Chlamydia* spp. (pathogens); γ-Proteobacteria such as *Buchnera* spp., *Wigglesworthia glossinidia brevipalpis,* and *Candidatus Blochmannia floridanus* (endosymbionts); α-Proteobacteria such as *Wolbachia* sp. (endosymbiont) and *R.* *prowazekii* (pathogen); Gram-positive-like organisms *Mycoplasma* spp. and *T. whipplei* (pathogens); Spirochetes such as *Tr. pallidum* and *Borrelia burgdorferi* (pathogens); and an archaeal symbiont, *Nanoarchaeum equitans*. Organisms in this cluster share an obligate intracellular lifestyle as well as reduced genome size. The clustering does not appear to be due to genome size alone as nonobligate intracellular organisms with small genome sizes do not fall into this cluster. One possibility is that the transport needs of these obligate intracellular organisms are more specialized than those of environmental organisms because of the much more static nature of their intracellular environments. This may have allowed them to shed, for example, transporters for alternative nitrogen/carbon sources, osmoregulatory functions, and ion homeostasis. Similar to their prokaryotic counterparts, two eukaryotic intracellular parasites, *P. falciparum* and *En. cuniculi,* form a distinct cluster separate from the other eukaryotes.

The soil/plant-associated microbe cluster also contains species from various phylogenetic groups, such as Actinobacteria *(Corynebacterium* and *Streptomyces),* Firmicutes *(Bacillus* and *Oceanobacillus),* α-Proteobacteria *(Brucella, Agrobacterium, Mesorhizobium, Sinorhizobium,* and *Bradyrhizobium),* β-Proteobacteria *(Bordetella* and *Ralstonia),* γ-Proteobacteria *(Pseudomonas* and *Rhodopseudomonas),* δ-Proteobacteria *(Geobacter),* Deinococcus *(Deinococcus radiodurans),* Planctomycetes (*Pirellula* sp.), and Bacteroidetes *(Bacteroides thetaiotaomicron).* All of these organisms possess a robust collection of transporter systems. It is unlikely that these species are merely clustered by their genome sizes because some species in this cluster have relatively smaller genome sizes, like *Corynebacterium efficiens* (3.0 Mb), *D. radiodurans* (3.2 Mb)*,* and *Brucella melitensis* (3.2 Mb). In addition, hierarchical clustering of organisms exclusively by genome size generates clusters with no apparent phylogenetic relationship (data not shown). The similarity of phylogenetic profiles of organisms in this cluster probably reflects the versatility of these organisms and their exposure to a wide range of different substrates in their natural environment. The majority of species in this cluster can be free-living in the soil, and some are capable of living in a diverse range of environments. They generally share a broad range of transport capabilities for plant-derived compounds specifically and for organic nutrients in general. Interestingly, some of the human pathogens, e.g., *Bordetella, Brucella, Bacillus anthracis* [26], and *Bacteroides thetaiotaomicron*, are also grouped in this cluster. All of these pathogens have close relatives that are soil- or plant-associated environmental organisms [49–52], so their transport capabilities probably reflect a combination of their evolutionary heritage, original environmental niche, and current transport needs.

To compare the transport capabilities of organisms in the intracellular pathogen/symbiont cluster and the soil/plant-associated microbe cluster, we carried out statistical analysis on their number of transporters, percentage of ORFs encoding transport proteins, and compositions in each transporter type (data not shown). Organisms in the soil/plant-associated microbe cluster on average have about eight times as many transporters as those in the intracellular organism cluster (*p* < 0.0001; *p-*value denotes the confidence level that the correlation observed is significantly different from the null hypothesis). The difference in the relative percentage of ORFs that are transporters is smaller but still significant (1.5-fold increase, *p* < 0.0001), suggesting that systematic gene loss and genome compaction is one of the important factors in reducing the number of transport proteins in intracellular organisms. The residual transport systems conserved in these obligate intracellular organisms probably belong to the core essential genes required for the acquisition of key nutrients and metabolic intermediates. For example, a glutamate transporter is encoded in two obligate endosymboints: the GltP glutamate:proton symporter (DAACS family) [53] in *Candidatus Blochmannia floridanus,* and GltJKL ABC transporter [54] in *Wigglesworthia glossinidia brevipalpis*. These organisms have a truncated citrate cycle that begins with α-ketoglutarate and ends with oxaloactetate [55]. Their citrate cycle could be closed by the transamination of the imported glutamate to aspartate, catalyzed by an aspartate aminotransferase (AspC) that uses oxaloactetate as a cosubstrate and produces α-ketoglutarate. As to the distribution of transporter types, there is no significant difference between these two clusters although intracellular organisms show a higher degree of variation in each transporter type than the plant/soil-associated microbes. These variations may reflect the unique internal environment inside the host cells. All these observations illustrate how adaptation of an organism to certain living conditions leads to changes in its transporter repertoire and at the same time determines the set of transporters that the organism cannot afford to lose.

In addition to investigating the relationship between organisms based on their transporter profiles, we also examined the clustering of transporter families. The essentially ubiquitous families, like ABC, MFS, P(F)-type ATPase, that are present in virtually every organism we analyzed, are clustered together. Eukaryotic-specific families, most of which are single-organism-specific ion channels, are grouped together. Interestingly, the sodium-ion-dependent families, like neurotransmitter:sodium symporter, alanine/glycine:cation symporter, solute:sodium symporter, and divalent anion:sodium symporter [56–58], are clustered together. Transporters in these families are all symporters that utilize the sodium ion gradient to transport amino acid, solute, and/or divalent ions into cytoplasm. This clustering may suggest that these families co-occur in a specific set of organisms, presumably those reliant on sodium-ion-driven transport.

Previous studies have shown that transporters with similar functions characteristically cluster together in phylogenetic analyses; hence, substrate specificity appears to be a conserved evolutionary trait in transporters [19,20,59,60]. The phylogenetic profiles of predicted substrates for all 141 organisms were generated and clustered by MeV (see Figure S3). Overall, similar patterns were observed as with the clustering by families. Organisms were grouped together either by their phylogenetic history or by their physiology or living habits. Ubiquitous substrates (e.g., cation, amino acid, sugar, and phosphate) and eukaryotic-specific substrates (e.g., cholesterol, UDP-sugars, and phosphoenolpyruvate) each form distinct clusters.

### Distribution of Transporter Families among Species in the Same Genus

**Figure 6:**
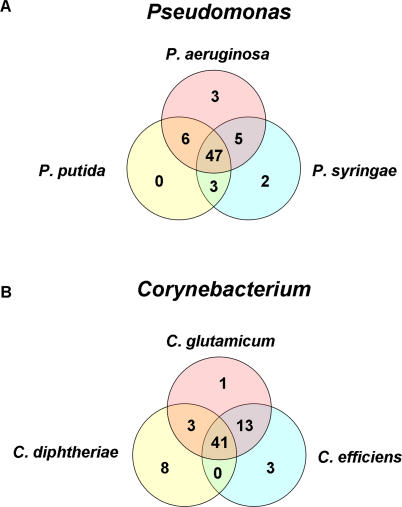
Venn Diagrams Showing the Distribution of Transporter Families among Species Belonging to the Same Genus (A) Transporter family distribution among three *Pseudomonas* species. (B) Transporter family distribution among three *Corynebacterium* species.

With the transporter data from a great diversity of sequenced organisms, we were able to compare the distribution of transporter families in closely related species (i.e., from the same genus) (Figures 6 and S4). In most of the cases we studied, species from the same genus share highly parallel distributions of transporter families. For example, three *Pseudomonas* species, *Ps. aeruginosa* [61], *Ps. putida* [62] and *Ps. syringae* [63], all of which are metabolically versatile soil/plant-associated bacteria, show highly similar patterns of transporter family distribution. Among the 66 transporter families present in this genus, 47 are shared by all three species and 14 are shared by two species (Figure 6A). All three species encode transporters for a diverse spectrum of substrates, including sugars, amino acids, peptides, carboxylates, and various cations and anions.

The distribution of transporter families in three *Corynebacterium* species represents an exception. *Co. glutamicum* [64] and *Co. efficiens* [65] are widely used in the industrial production of amino acids like glutamic acid and lysine by fermentation. The closely related *Co. diphtheriae* [66], however, is a human pathogen causing the respiratory illness diphtheria and lacks amino acid productivity. Compared to the other two species, *Co. diphtheriae* shows a dramatically different transporter family profile (Figure 6B). There are eight families specific to *Co. diphtheriae,* while only one for *Co. glutamicum* and three for *Co. efficiens*. More importantly, *Co. diphtheriae* uses totally different mechanisms to transport potassium ion and C4-dicarboxylates than the other two species. In *Co. diphtheriae,* potassium ions are transported into cytoplasm via a Trk family K^+^:H^+^ symporter [67], while both *Co. glutamicum* and *Co. efficiens* encode a KUP family potassium ion uptake permease [68]. *Co. diphtheriae* utilizes the DcuABC antiporter system [69] for the uptake of C4-dicarboxylate, while the other species use the ATP-independent tripartite periplasmic symporter systems (TRAP-T family) [70]. The common orthologs of transporters in families specific to one or two *Corynebacterium* species were identified in sequenced high-GC Gram-positive bacteria, and the phylogenetic trees were constructed by the neighbor-joining method (data not shown). For those families with orthologs in *Co. glutamicum* and *Co. efficiens* but not in *Co. diphtheriae,* orthologs were also identified in the majority of high-GC Gram-positive species. The trees of transport protein are similar to the 16S rRNA tree, suggesting certain transporter families in *Co. efficiens* are missing because of specific gene losses. By contrast, *Co. diphtheriae*–specific transporter families, like Dcu, DcuC, and Trk families, tend to have either no apparent orthologs or only distantly related homologs in other sequenced high-GC Gram-positive species, suggesting possible evolutionary gene acquisition events in *Co. diphtheriae*. The recent finding that both gene loss and horizontal gene transfer are responsible for the functional differentiation in amino acid biosynthesis of the three *Corynebacterium* species [71] further supports this conclusion.

All three *Corynebacterium* species share 41 transporter families. Interestingly, although *Co. diphtheriae* shows no amino acid productivity and has a reduced genome size [71], all the major types of amino acid exporters in *Co. glutamicum* [72] are conserved in *Co. diphtheriae*, e.g., the LysE family transporter for the export of basic amino acids, the RhtB family transporter for threonine efflux, the ThrE family transporter for threonine and serine export, and the LIV-E family transporter (BrnFE in *Co. glutamicum*), which is a two-component efflux pump exporting branched-chain amino acids [73]. The only difference observed among these organisms is the number of paralogs in the RhtB family: three in *Co. glutamicum,* two in *Co. efficiens,* and only one in *Co. diphtheriae*. The phylogenetic tree of the RhtB family suggests that gene duplication took place in the common ancestor of *Corynebacterium,* and that specific gene loss was responsible for the single RhtB transporter in *Co. diphtheriae*.

### Conclusion

The rapid expansion of complete genome sequencing enabled us to conduct analyses of transporter capabilities on the whole-genome level. By comparing the membrane transport systems in Eubacteria, Archaea, and Eukaryota, we could draw conclusions as follows. (1) Eukaryotic species generally encode a larger number of transporters, but transporters account for a smaller percentage of total ORFs in eukaryotic than in prokaryotic species. Prokaryotic obligate intracellular pathogens and endosymbionts, as well as the eukaryotic parasites, possess the most limited repertoire of membrane transporters. (2) Organisms with a larger genome size tend to have a higher number of transporters. In prokaryotes and unicellular eukaryotes, this increase is primarily due to increased diversity of types of transporter. In multicellular eukaryotes, this increase is largely due to the greater number of paralogs by gene duplication or expansion in certain transporter families. (3) The distribution of different transporter types according to transport mode and energy coupling mechanism generally correlates with organisms' primary mechanism of energy generation. Compared to eukaryotes, prokaryotic species rely heavily on primary (active) transporters. Primary type transporters in Eubacteria and Archaea account for a much larger percentage of total transporters than any other transporter type. This phenomenon may be related to the absence of ABC-type uptake permeases in eukaryotes and, in some cases, the bioenergetic requirements and environmental constraints of prokaryotic organisms. (4) Energy-independent channel proteins are far more numerous in multicellular organisms and are often involved in cell–cell communication and signal transduction processes. Many channels are restricted to a single organismal type. The expression of different subunits of a channel in a timely fashion may be an essential step during embryonic development in mammals. (5) The PTS is only present in a subset of Eubacteria, and is completely absent in Archaea and Eukaryota. The expansion of sugar PTS systems in species dwelling in the gastrointestinal tract could provide the advantage to thrive in their ecological niches. (6) Hierarchical clustering of the phylogenetic profiles of transporter families showed that the distribution of transporter families appears to reflect a combination of evolutionary history and environment and lifestyle factors. (7) The distribution pattern of transporter families in species belonging to the same genus is usually parallel, with some notable exceptions that may reflect specific environmental differences.

## Materials and Methods

We developed a semi-automated pipeline to annotate transport systems genome-wide, input the data into TransportDB database, and visualize the result through a Web interface [74]. The complete protein sequences from specific organisms were first searched against our curated database of transport proteins for similarity to known or putative transport proteins using BLAST [75,76]. All of the query proteins with significant hits (*E*-value < 0.001) were collected and searched against the NCBI nonredundant protein database and Pfam database [77]. Transmembrane protein topology was predicted by TMHMM [78]. A Web-based interface was created to facilitate the annotation processes, which incorporates number of hits to the transporter database, BLAST and HMM search *E*-value and score, number of predicted transmembrane segments, and the description of top hits to the nonredundant protein database. We also set up direct links between transporter classification family and COG classification [79] so that COG-based searches can inform the transporter annotation. The results can be viewed at the TransportDB Web site (http://www.membranetransport.org/).

To analyze the phylogenetic profiles of transporter families and predicted substrates, we assigned a profile to each transporter family or substrate. Each profile is a string with 141 entries (number of species analyzed). If a given family is present or a given substrate is transported in certain species, the value one was assigned at these positions (red for transporter families/purple for predicted substrates). If not, zero was assigned (black). Transporter families or substrates were clustered according to the similarity of their phylogenetic profiles using The Institute for Genomic Research's microarray multi-experiment viewer (MeV) [80] with two-dimensional hierarchical clustering as described by Eisen et al. [81].

## Supporting Information

Figure S1 Comparison of the Percentage of Membrane Proteins with Six or More Transmembrane Segments That Were Annotated as “Hypothetical Protein” in Selected Archaea and Eubacteria (37 KB PDF)

Figure S2 Detailed View of the Hierarchical Clustering of Phylogenetic Profiles of Transporter Families (A) Clustering of species.
(B) Clustering of transporter families.
(722 KB PPT)

Figure S3 Phylogenetic Profiling of Predicted Transporter Substrates Phylogenetic profiles were created for each predicted substrate. Each profile is a string with 141 entries (number of organisms analyzed). If a specific substrate is transported in a given organism, the value one is assigned at this position (purple). If not, zero is assigned (black). Organisms and substrates were clustered according to the similarity of their phylogenetic profiles.
(671 KB PDF)

Figure S4 Venn Diagrams Showing the Distribution of Transporter Families among Species Belonging to the Same Genus (A) Transporter family distribution among three *Bordetella* species.
(B) Transporter family distribution among three *Chlamydia* species.
(C) Transporter family distribution among three *Mycobacterium* species.
(D) Transporter family distribution among three *Pyrococcus* species.
(E) Transporter family distribution among three *Streptococcus* species.
(F) Transporter family distribution among three *Vibrio* species.
(947 KB PDF)

Table S1 List of 141 Organisms Analyzed in This Study (194 KB DOC)

## Abbreviations

ABC: adenosine triphosphate–binding cassette; ATP, adenosine triphosphate
GIC: glutamate-gated ion channel
MFS: major facilitator superfamily
ORF: open reading frame
PTS: phosphotransferase system
