# Supplementary material for: Comparative Analyses of Fundamental Differences in Membrane Transport Capabilities in Prokaryotes and Eukaryotes
Source: PLoS Comput Biol. 2005 Aug 19;1(3):e27. doi: 10.1371/journal.pcbi.0010027 (PMC1188273; doi:10.1371/journal.pcbi.0010027)
Supplement: Supplementary file 1 [file pcbi.0010027.sg001.pdf]

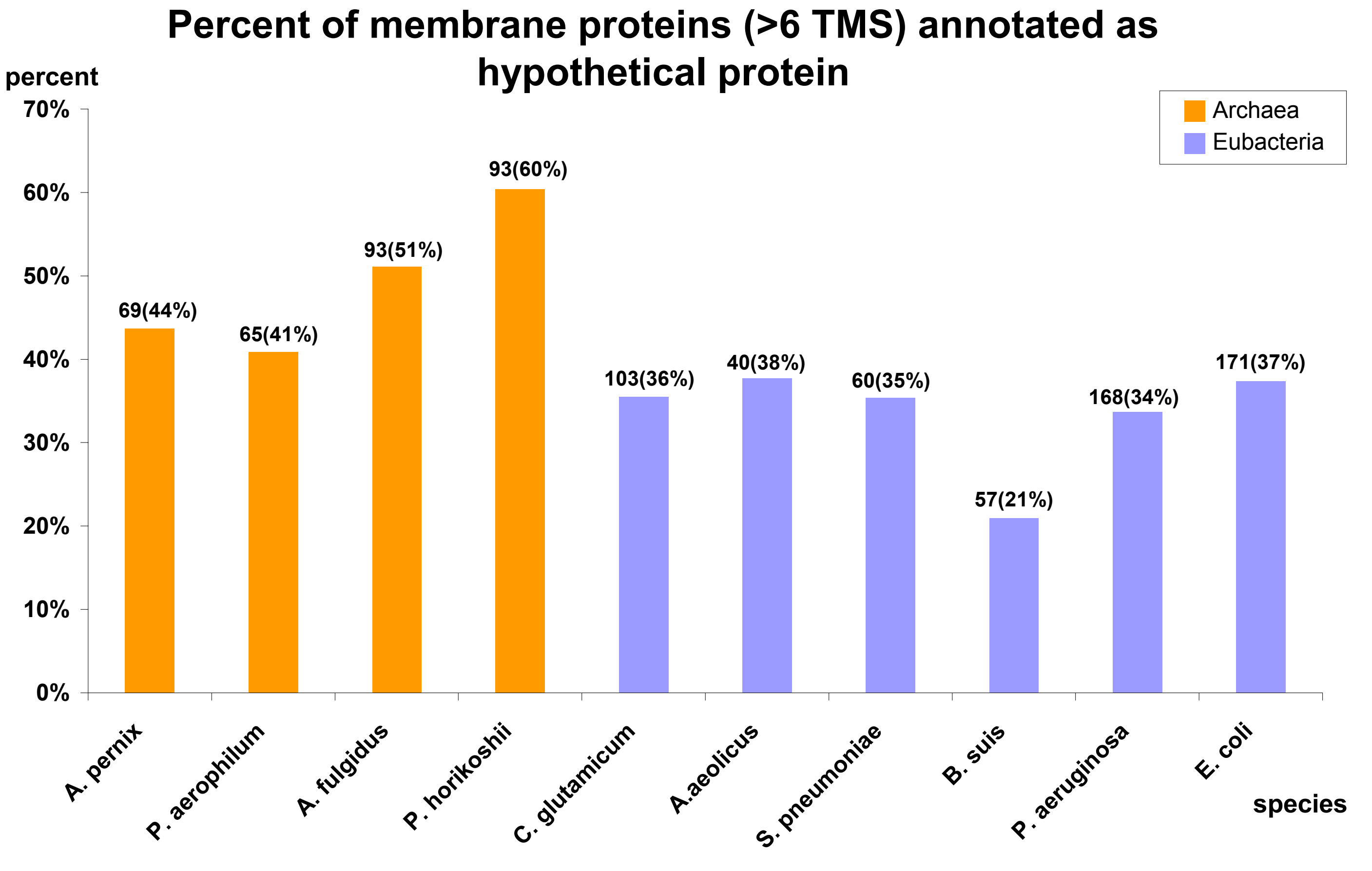
Percent of membrane proteins (>6 TMS) annotated as hypothetical protein
percent
70%
60%
50%
40%
30%
20%
10%
0%
Archaea
Eubacteria
69(44%)
65(41%)
93(51%)
93(60%)
103(36%)
40(38%)
60(35%)
57(21%)
168(34%)
171(37%)
A. pernix
P. aerophilum
A. fulgidus
P. horikoshii
C. glutamicum
A.aeolicus
S. pneumoniae
B. suis
P. aeruginosa
E. coli
species
