# Supplementary material for: Comparative Analyses of Fundamental Differences in Membrane Transport Capabilities in Prokaryotes and Eukaryotes
Source: PLoS Comput Biol. 2005 Aug 19;1(3):e27. doi: 10.1371/journal.pcbi.0010027 (PMC1188273; doi:10.1371/journal.pcbi.0010027)

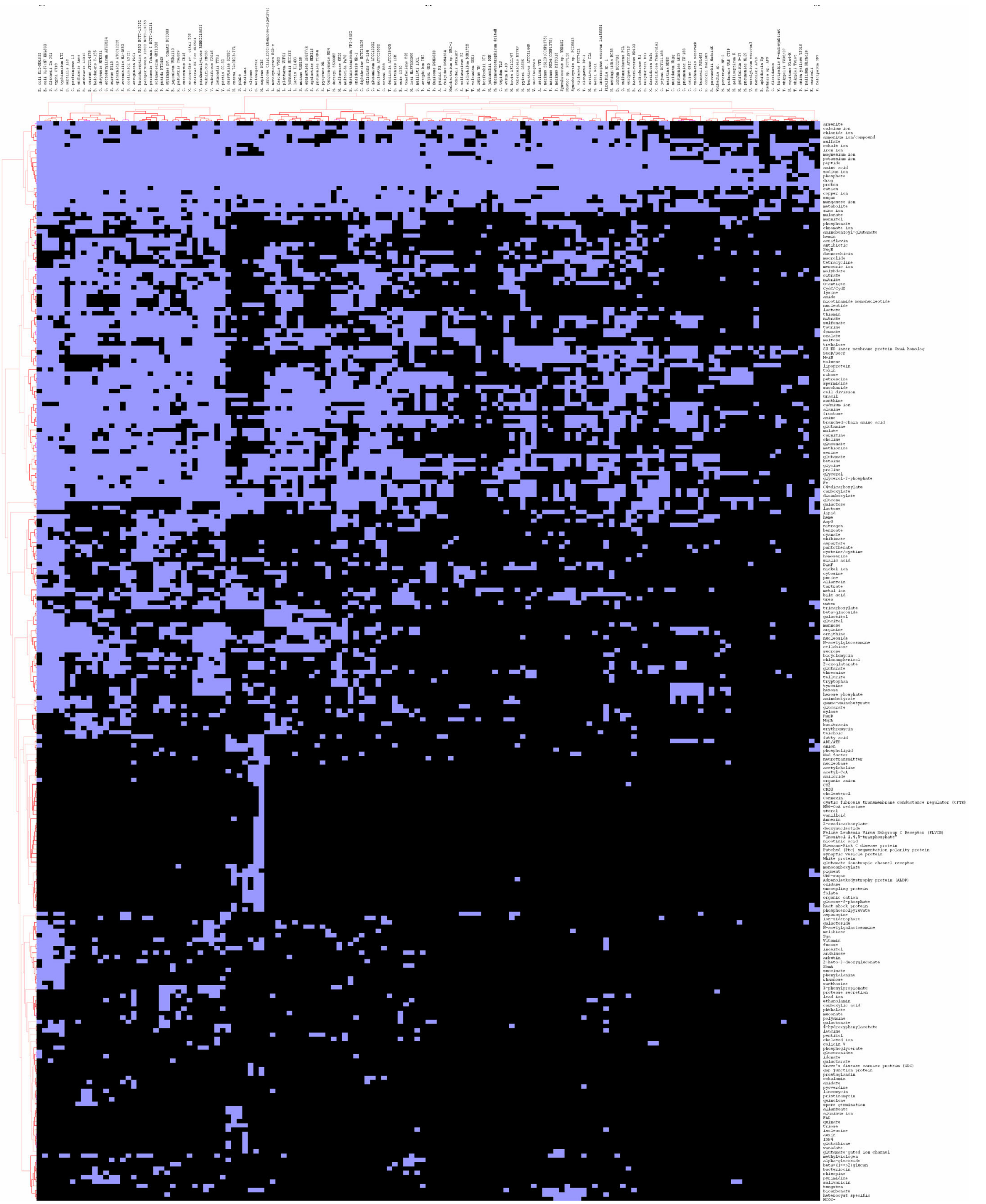

Supplement: Supplementary file 2 [file pcbi.0010027.sg003.pdf]
