# Supplementary material for: Comparative Analyses of Fundamental Differences in Membrane Transport Capabilities in Prokaryotes and Eukaryotes
Source: PLoS Comput Biol. 2005 Aug 19;1(3):e27. doi: 10.1371/journal.pcbi.0010027 (PMC1188273; doi:10.1371/journal.pcbi.0010027)
Supplement: Supplementary file 3 [file pcbi.0010027.sg004.pdf]

A

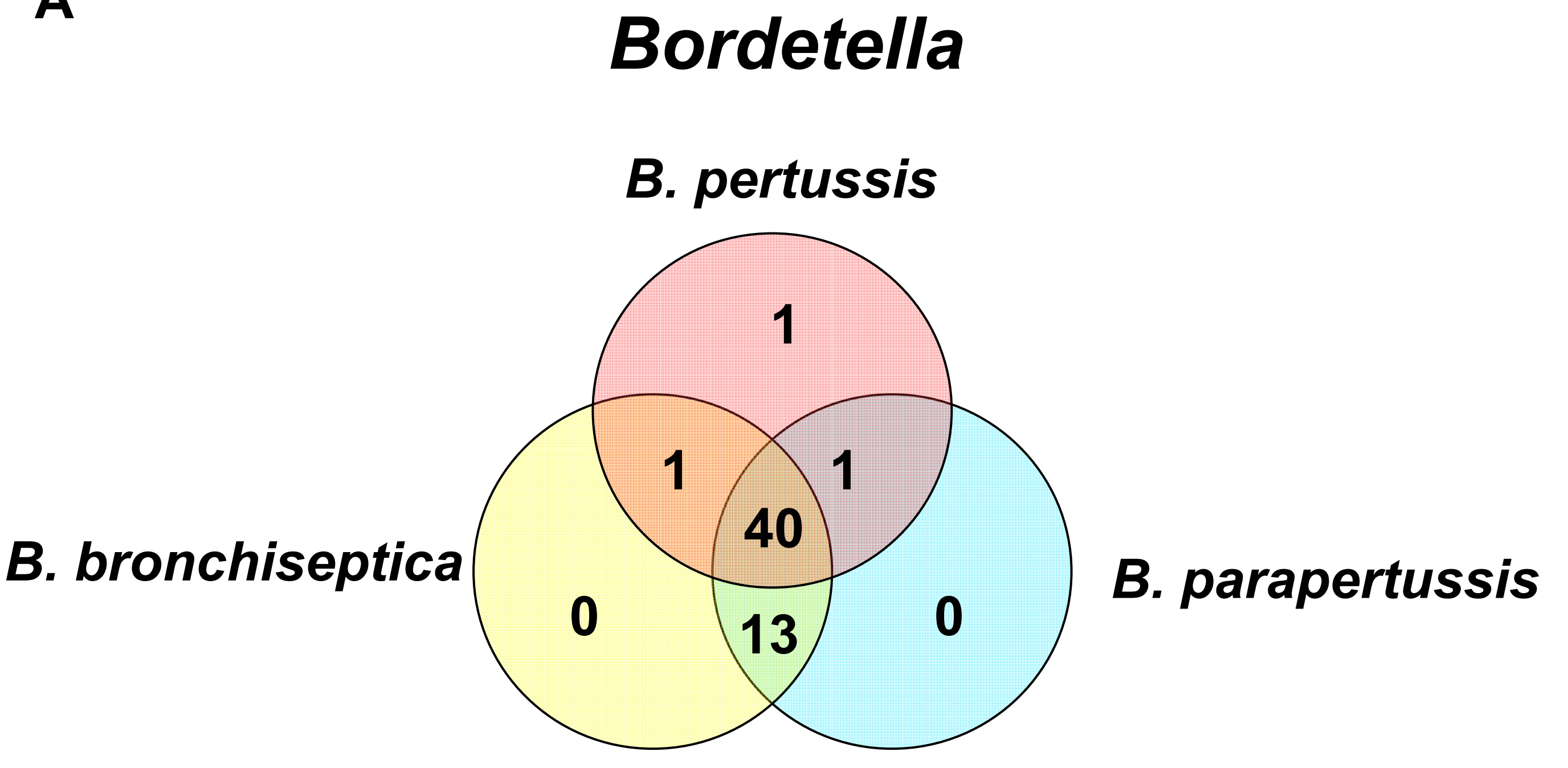
Bordetella
B. pertussis
B. bronchiseptica
B. parapertussis
1
1
1
40
0
13
0

B

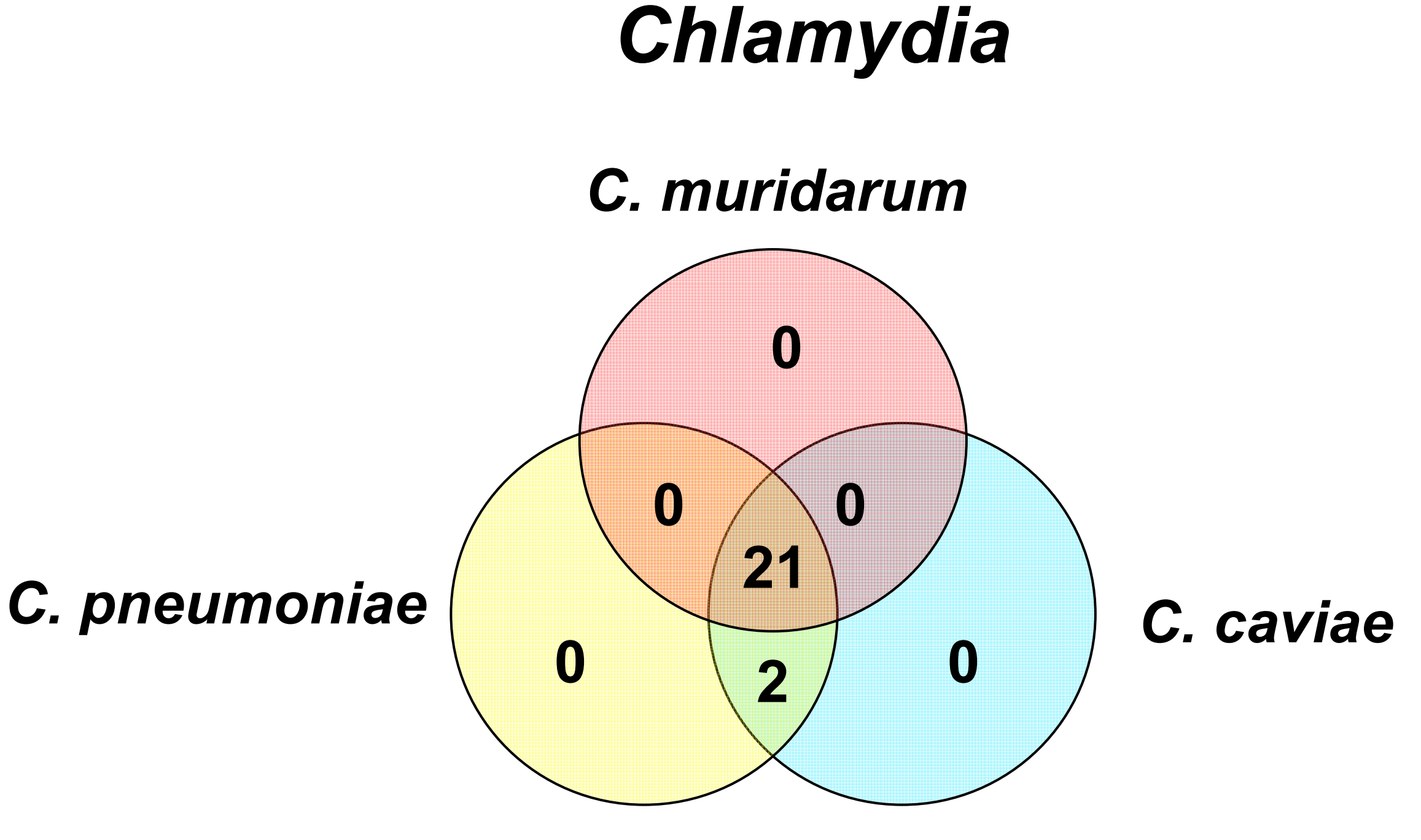
Chlamydia
C. muridarum
C. pneumoniae
C. caviae
0
0
0
21
0
2
0

C

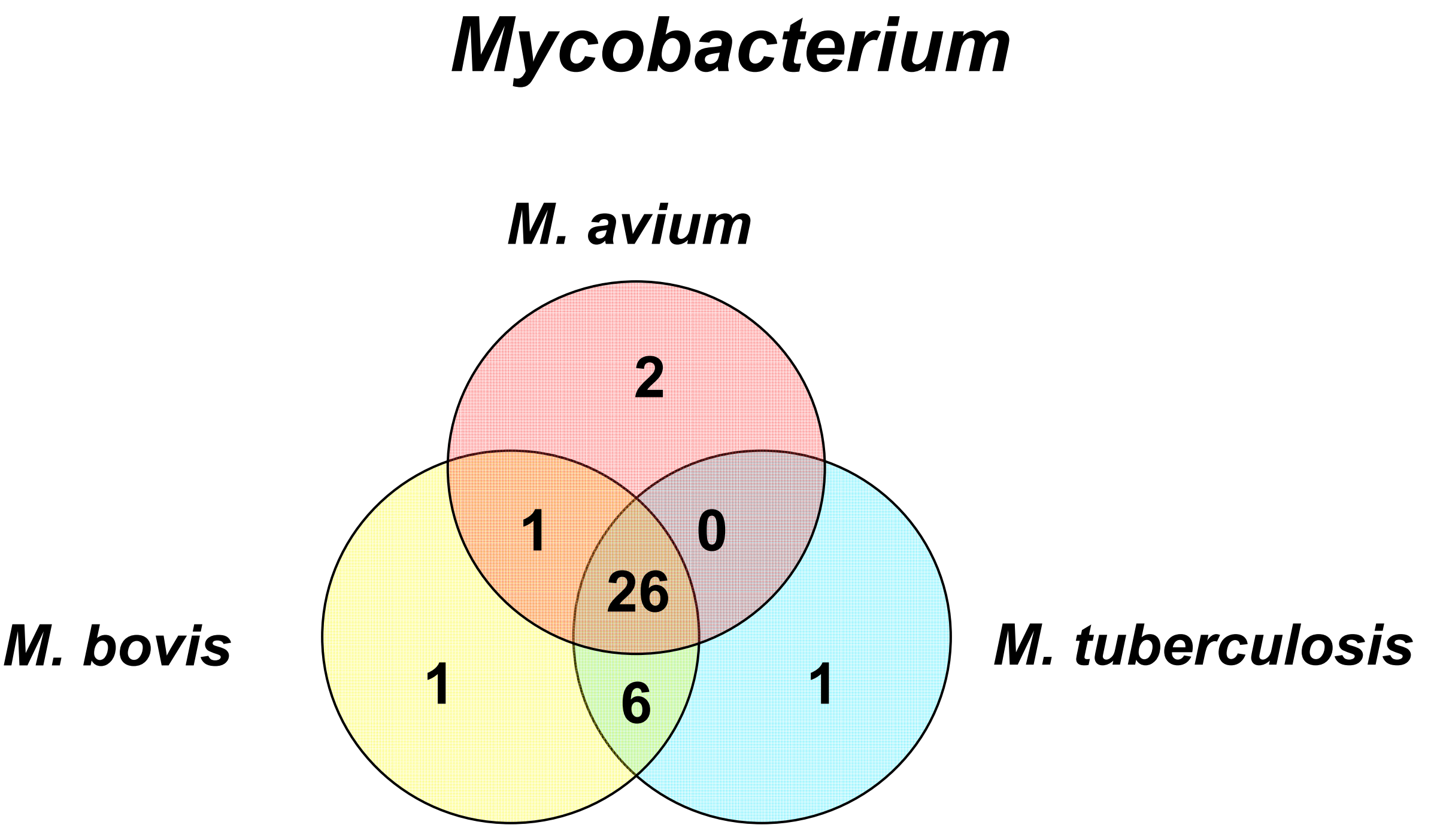
Mycobacterium
M. avium
M. bovis
M. tuberculosis
2
1
0
26
1
6
1

D

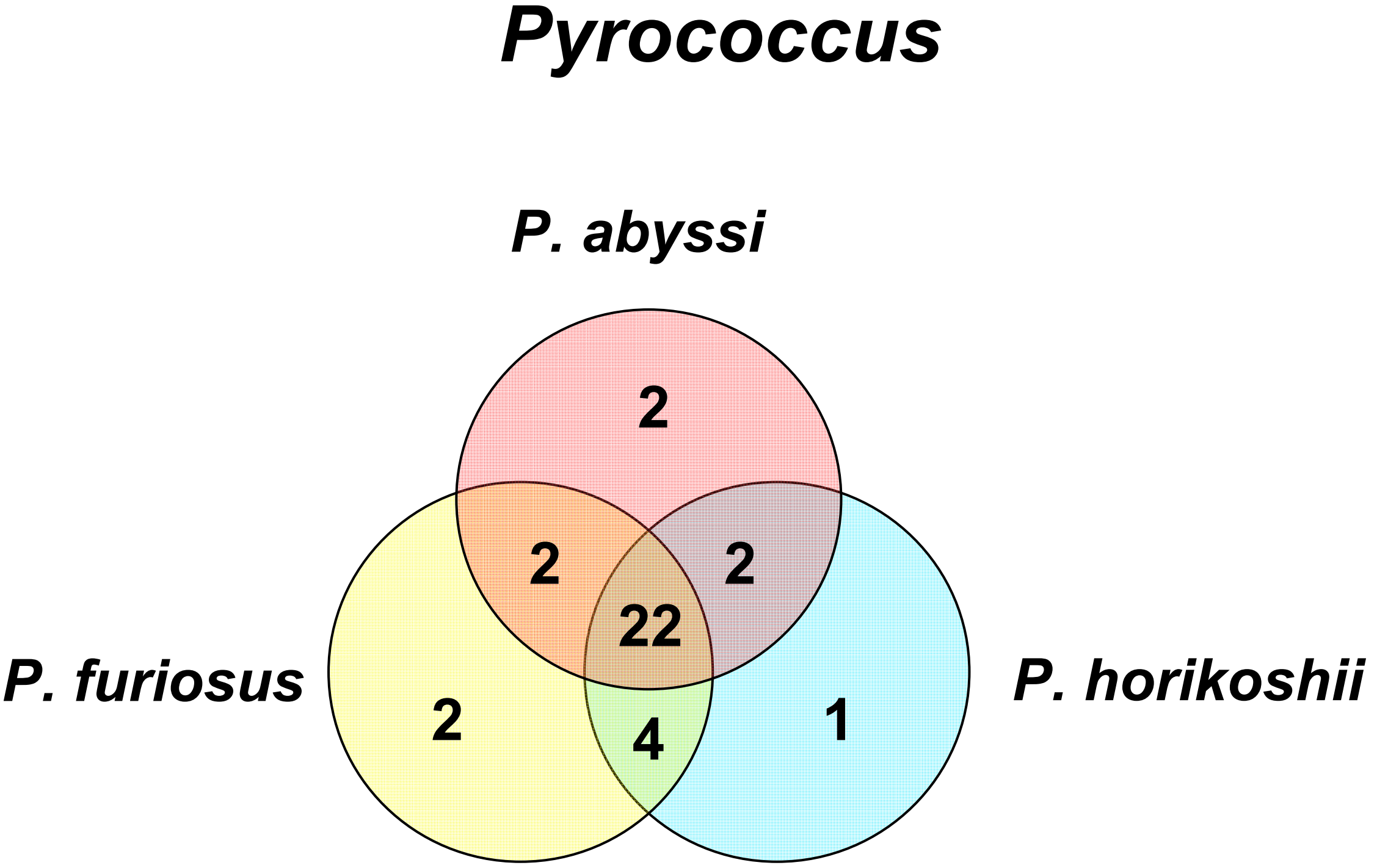
Pyrococcus
P. abyssi
P. furiosus
P. horikoshii
2
2
2
22
2
4
1

E

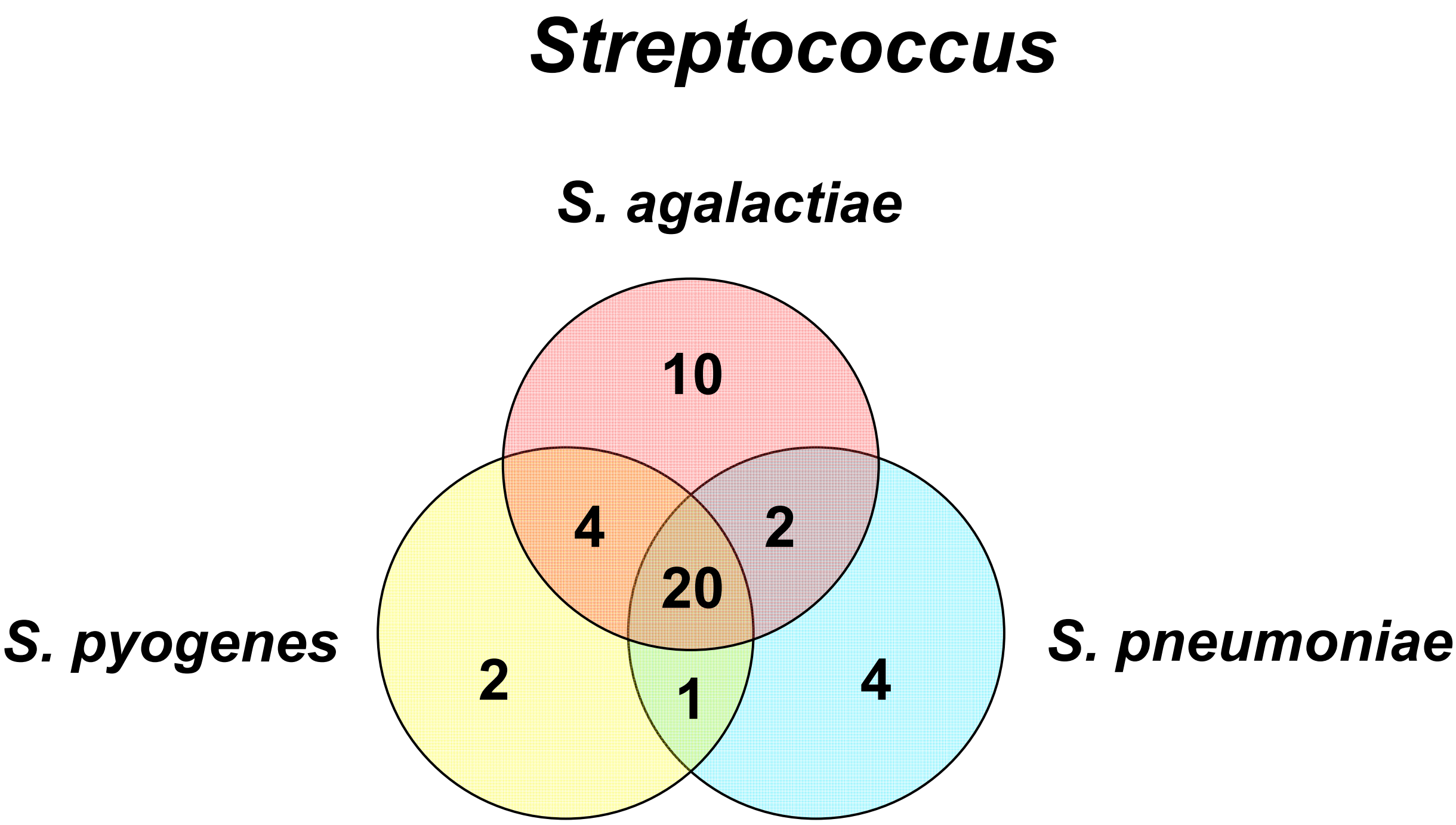
Streptococcus
S. agalactiae
S. pyogenes
S. pneumoniae
10
4
2
20
2
1
4

F

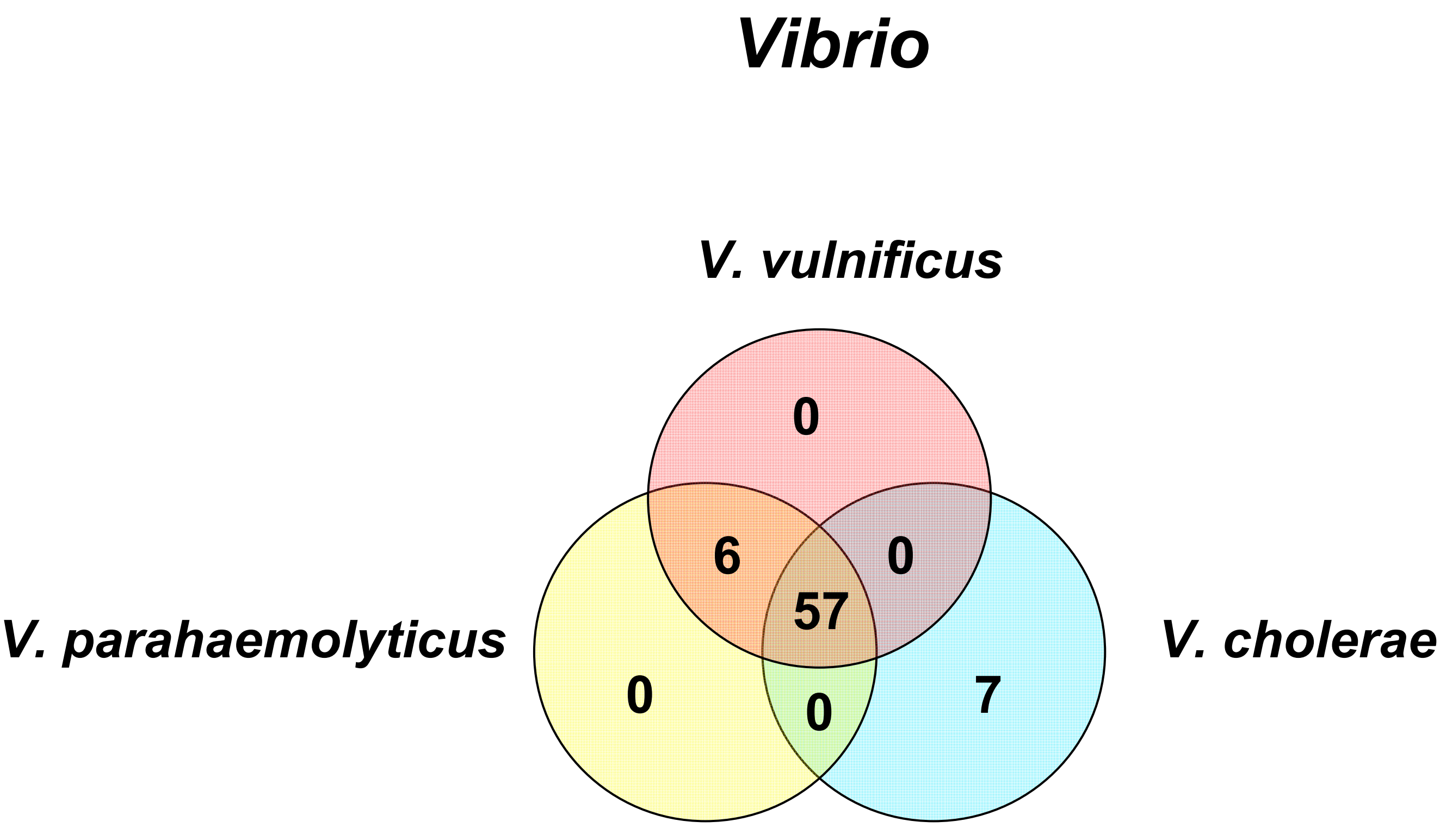
Vibrio
V. vulnificus
V. parahaemolyticus
V. cholerae
0
6
0
57
0
0
7
