# Supplementary material for: Comparative Analyses of Fundamental Differences in Membrane Transport Capabilities in Prokaryotes and Eukaryotes
Source: PLoS Comput Biol. 2005 Aug 19;1(3):e27. doi: 10.1371/journal.pcbi.0010027 (PMC1188273; doi:10.1371/journal.pcbi.0010027)
Supplement: Supplementary file 4 [file pcbi.0010027.st001.doc]

|  | **Taxonomy** | **Species** |
| --- | --- | --- |
| **Archaea** | Crenarchaeota | *Aeropyrum pernix K1* |
|  |  | *Pyrobaculum aerophilum IM2* |
|  |  | *Sulfolobus solfataricus P2* |
|  |  | *Sulfolobus tokodaii strain7* |
|  | Euryarchaeota | *Archaeoglobus fulgidus DSM4304* |
|  |  | *Halobacterium sp. NRC-1* |
|  |  | *Methanobacterium thermoautotrophicum deltaH* |
|  |  | *Methanococcus jannaschii DSM* |
|  |  | *Methanopyrus kandleri AV19* |
|  |  | *Methanosarcina acetivorans C2A* |
|  |  | *Methanosarcina mazei Goe1* |
|  |  | *Pyrococcus abyssi GE5* |
|  |  | *Pyrococcus furiosus DSM3638* |
|  |  | *Pyrococcus horikoshii OT3* |
|  |  | *Thermoplasma acidophilum DSM1728* |
|  |  | *Thermoplasma volcanium GSS1* |
|  | Nanoarchaea | *Nanoarchaeum equitans Kin4-M* |
| **Eubacteria** | Actinobacteria | *Bifidobacterium longum NCC2705* |
|  |  | *Corynebacterium efficiens YS-314* |
|  |  | *Corynebacterium glutamicum ATCC13032* |
|  |  | *Mycobacterium leprae TN* |
|  |  | *Mycobacterium tuberculosis H37Rv* |
|  |  | *Mycobacterium avium K-10* |
|  |  | *Mycobacterium bovis AF2122/97* |
|  |  | *Streptomyces avermitilis MA-4680* |
|  |  | *Streptomyces coelicolor A3(2)* |
|  |  | *Tropheryma whipplei Twist* |
|  |  | *Corynebacterium diphtheriae NCTC13129* |
|  |  | *Tropheryma whippelii TW08/27* |
|  | Aquificae | *Aquifex aeolicus VF5* |
|  | Bacteroidetes | *Bacteroides thetaiotaomicron VPI-5482* |
|  |  | *Chlorobium tepidum TLS* |
|  | Chlamydia | *Chlamydia muridarum Nigg* |
|  |  | *Chlamydia pneumoniae AR39* |
|  |  | *Chlamydia trachomatis serovarD* |
|  |  | *Chlamydophila caviae GPIC* |
|  |  | *Chlamydophila pneumoniae TW-183* |
|  | Cyanobacteria | *Gloeobacter violaceus PCC7421* |
|  |  | *Nostoc sp. PCC7120* |
|  |  | *Prochlorococcus marinus MIT9313* |
|  |  | *Prochlorococcus marinus SS120(CCMP1375)* |
|  |  | *Prochlorococcus marinus MED4(CCMP1378)* |
|  |  | *Synechococcus sp. WH8102* |
| **Eubacteria** |  | *Synechocystis sp. PCC6803* |
|  |  | *Thermosynechococcus elongatus BP-1* |
|  | Deinococcus-Thermus | *Deinococcus radiodurans R1* |
|  | Firmicutes | *Bacillus anthracis Ames* |
|  |  | *Bacillus cereus ATCC14579* |
|  |  | *Bacillus halodurans C-125* |
|  |  | *Bacillus subtilis 168* |
|  |  | *Clostridium acetobutylicum ATCC824* |
|  |  | *Bacillus anthracis A2012* |
|  |  | *Clostridium perfringens 13* |
|  |  | *Clostridium tetani E88* |
|  |  | *Enterococcus faecalis V583* |
|  |  | *Lactobacillus plantarum WCFS1* |
|  |  | *Lactobacillus johnsonii NCC533* |
|  |  | *Lactococcus lactis IL1403* |
|  |  | *Listeria innocua Clip11262(rhamnose-negative)* |
|  |  | *Listeria monocytogenes EGD-e* |
|  |  | *Mycoplasma gallisepticum R* |
|  |  | *Mycoplasma genitalium G-37* |
|  |  | *Mycoplasma penetrans HF-2* |
|  |  | *Mycoplasma pneumoniae M129* |
|  |  | *Mycoplasma pulmonis UAB CTIP* |
|  |  | *Oceanobacillus iheyensis HTE831* |
|  |  | *Phytoplasma onion yellows YJ016* |
|  |  | *Staphylococcus aureus N315* |
|  |  | *Staphylococcus epidermidis ATCC12228* |
|  |  | *Streptococcus agalactiae 2603V/R* |
|  |  | *Streptococcus agalactiae NEM316* |
|  |  | *Streptococcus mutans UAB159* |
|  |  | *Streptococcus pneumoniae TIGR4* |
|  |  | *Streptococcus pyogenes M1* |
|  |  | *Thermoanaerobacter tengcongensis MB4* |
|  |  | *Ureaplasma urealyticum serovar3* |
|  | Fusobacteria | *Fusobacterium nucleatum ATCC25586* |
|  | Planctomycetes | *Pirellula sp. 1* |
|  | Proteobacteria-Alpha | *Agrobacterium tumefaciens C58* |
|  |  | *Bradyrhizobium japonicum USDA110* |
|  |  | *Brucella melitensis 16M* |
|  |  | *Brucella suis 1330* |
|  |  | *Caulobacter crescentus CB15* |
|  |  | *Mesorhizobium loti MAFF303099* |
|  |  | *Rickettsia conorii Malish7* |
|  |  | *Rickettsia prowazekii MadridE* |
|  |  | *Sinorhizobium meliloti 1021* |
| **Eubacteria** |  | *Wolbachia sp.* |
|  | Proteobacteria-Beta | *Bordetella bronchiseptica RB50 NCTC-13252* |
|  |  | *Bordetella parapertussis 12822 NCTC-13253* |
|  |  | *Bordetella pertussis Tohama I NCTC-13251* |
|  |  | *Neisseria meningitidis MC58* |
|  |  | *Nitrosomonas europaea ATCC19718* |
|  |  | *Ralstonia solanacearum GMI1000* |
|  | Proteobacteria-Delta | *Bdellovibrio bacteriovorus HD100* |
|  |  | *Geobacter sulfurreducens PCA* |
|  | Proteobacteria-Epsilon | *Campylobacter jejuni NCTC11168* |
|  |  | *Helicobacter pylori 26695* |
|  |  | *Helicobacter hepaticus ATCC51449* |
|  |  | *Wolinella succinogenes* |
|  | Proteobacteria-Gamma | *Buchnera aphidicola Sg* |
|  |  | *Buchnera sp. APS* |
|  |  | *Candidatus Blochmannia floridanus* |
|  |  | *Coxiella burnetii RSA493* |
|  |  | *Escherichia coli K12-MG1655* |
|  |  | *Escherichia coli O157:H7 EDL933* |
|  |  | *Haemophilus influenzae KW20* |
|  |  | *Haemophilus ducreyi 35000HP* |
|  |  | *Pasteurella multocida Pm70* |
|  |  | *Photorhabdus lumines laumondii TTO1* |
|  |  | *Pseudomonas aeruginosa PAO1* |
|  |  | *Pseudomonas putida KT2440* |
|  |  | *Pseudomonas syringae pv. tomato DC3000* |
|  |  | *Rhodopseudomonas palustris CGA009* |
|  |  | *Salmonella typhi CT18* |
|  |  | *Salmonella typhimurium LT2* |
|  |  | *Shewanella oneidensis MR-1* |
|  |  | *Shigella flexneri 2a 301* |
|  |  | *Vibrio cholerae El Tor N16961* |
|  |  | *Vibrio parahaemolyticus RIMD2210633* |
|  |  | *Vibrio vulnificus CMCP6* |
|  |  | *Vibrio vulnificus YJ016* |
|  |  | *Wigglesworthia brevipalpis P-endosymbiont* |
|  |  | *Xanthomonas axonopodis pv. citri 306* |
|  |  | *Xylella fastidiosa 9a5c* |
|  |  | *Xylella fastidiosa Temecula1* |
|  |  | *Yersinia pestis CO-92* |
|  | Spirochaetes | *Borrelia burgdorferi B31* |
|  |  | *Leptospira interrogans serovar lai56601* |
|  |  | *Treponema denticola ATCC35405* |
|  |  | *Treponema pallidum Nichols* |
|  | Thermotogales | *Thermotoga maritima MSB8* |
| **Eukaryota** | Fungi | *Neurospora crassa 74-OR23-IVA* |
|  |  | *Saccharomyces cerevisiae S288C* |
|  |  | *Schizosaccharomyces pombe* |
|  | Insects | *Drosophila melanogaster* |
|  | Microsporidia | *Encephalitozoon cuniculi* |
|  | Nematodes | *Caenorhabditis elegans* |
|  | Plants | *Arabidopsis thaliana* |
|  | Primates | *Homo sapiens* |
|  | Protozoa | *Plasmodium falciparum 3D7* |
